# *In vivo* molecular targeted radiotherapy

**DOI:** 10.2349/biij.1.2.e9

**Published:** 2005-10-01

**Authors:** AC Perkins

**Affiliations:** Academic Medical Physics, Medical School, Queen's Medical Centre, Nottingham, United Kingdom

**Keywords:** Radionuclide therapy, nuclear medicine therapy, targeted therapy

## Abstract

Unsealed radionuclides have been in clinical therapeutic use for well over half a century. Following the early inappropriate clinical administrations of radium salts in the early 20th century, the first real clinical benefits became evident with the use of ^131^I-sodium iodide for the treatment of hypothyroidism and differentiated thyroid carcinoma and ^32^P-sodium phosphate for the treatment of polycythaemia vera. In recent years the use of bone seeking agents ^89^Sr, ^153^Sm and ^186^Re for the palliation of bone pain have become widespread and considerable progress has been evident with the use of ^131^I-MIBG and ^90^Y-somatostatin receptor binding agents. Although the use of monoclonal antibody based therapeutic products has been slow to evolve, the start of the 21st century has witnessed the first licensed therapeutic antibody conjugates based on ^90^Y and ^131^I for the treatment of non-Hodgkin's lymphoma. The future clinical utility of this form of therapy will depend upon the development of radiopharmaceutical conjugates capable of selective binding to molecular targets. The availability of some therapeutic radionuclides such as ^188^Re produced from the tungsten generator system which can produce activity as required over many months, may make this type of therapy more widely available in some remote and developing countries.

Future products will involve cytotoxic radionuclides with appropriate potency, but with physical characteristics that will enable the administration of therapeutic doses with the minimal need for patient isolation. Further developments are likely to involve molecular constructs such as aptamers arising from new developments in biotechnology.

Patient trials are still underway and are now examining new methods of administration, dose fractionation and the clinical introduction of alpha emitting radiopharmaceutical conjugates. This review outlines the history, development and future potential of these forms of therapy.

## INTRODUCTION

Nuclear medicine therapy is based on the use of potent radionuclides normally attached to a ligand or incorporated into a conjugate and administered to patients as unsealed radioactive sources. The underlying principle is based on the selective molecular uptake of the agent in the lesion to be treated where it releases its cytotoxic dose of radiation with minimal uptake in normal tissues. This form of treatment is often referred to as targeted radiotherapy, however the term “molecular targeted therapy” would be more appropriate.

Initial interest in radionuclide therapy began during the early years of the 20th century with the inappropriate systemic use of radium salts at a time when the early radiologists considered radioactivity to be a “God given gift” with natural energising properties that could be used for the benefit of mankind. ^226^Radium was first isolated by the Curies in 1898 and was mainly used in the form of radium bromide, chloride, sulphate or carbonate. These early radium treatments were often based on no more than a solution for injection, drinking or bathing or as an ointment for topical application. In 1914 the title page of the journal “Radium” contained an article on the influence of intravenous injection of soluble radium salts in high blood pressure [1]. Despite the lack of clinical evidence, radium treatments were claimed to have benefit in the treatment of a range of conditions including arthritis, gout, neuralgia, lumbago, menstrual irregularities, sexual disorders and obesity!

Following the production of artificial radioactivity in the 1930s and 40s the first clinical treatments with ^32^P and ^131^I were undertaken. In particular the use of iodine for the treatment of thyrotoxicosis and thyroid cancer laid down the foundations for modern nuclear medicine. As the science of nuclear medicine developed and the clinical evidence base grew there has been a growing interest in a range of radionuclides for targeted molecular radiotherapy. However, progress has been slow whilst radiochemists, physicists and clinicians have worked on the design and development of radionuclide conjugates for new and novel treatments.

## THERAPEUTIC RADIONUCLIDES

Three principle factors affect the suitability of a radionuclide for potential therapeutic use. These are the physical, chemical and biological properties. For therapy the biological effect is dependent upon the emission of potent radiation such as beta particles with high loss of energy to the surrounding medium i.e. high Linear Energy Transfer (LET). The energy of the emitted radiation is directly proportional to the path length of the dose deposition in tissue. This can be used to give an indication of the most appropriate size of lesion for any given radionuclide. Using this information Wheldon *et al.* [2] showed that ^131^I with a mean beta path length of 0.9 mm was most suited to treating 3 mm size tumours whereas the more energetic beta emissions from ^90^Y would be more effective for the treatment of a 2 cm diameter tumour. One particular feature of this approach is that unlike conventional chemotherapy the radiopharmaceutical does not have to be incorporated into every tumour cell to have a therapeutic effect. The path length of the emitted radiation is sufficient to allow effective therapy following uptake into a subpopulation of cells. This is generally known as the “Bystander Effect”. A list of some of the main therapeutic radionuclides is given in Table 1.

**Table 1:** Physical characteristics of some therapeutic radionuclides

Nuclide	Physical half-life	Emission	Mean path length
			
^125^I	60.0d	auger	10 nm
^221^At	7.2h	alpha	65 nm
^213^Bi	46min	alpha	80 nm
^177^L	6.7d	beta/gamma	0.7 mm
^67^Cu	2.58d	beta/gamma	0.7 mm
^131^I	8.04d	beta/gamma	0.9 mm
^153^Sm	1.95d	beta/gamma	1.2 mm
^186^Re	3.8d	beta/gamma	1.8 mm
^32^P	14.3d	beta	2.9 mm
^188^Re	17h	beta/gamma	3.5 mm
^114m^In	50d	beta/gamma	3.6 mm
^90^Y	2.67d	beta	3.9 mm
			

For radionuclide imaging one of the main desirable requirements is for the radiopharmaceutical to contain a radionuclide that is a pure gamma emitter or at least having little particle emission (e.g. beta or alpha) thus keeping unnecessary radiation burden to the patient to a minimum. The converse is true for therapy since the presence of high-energy gamma emission from a therapeutic radiopharmaceutical will have the potential for unnecessary exposure to staff and relatives. As a result the patients require isolation after administration of the therapeutic agent to minimise exposure to other individuals. This is the case with radioiodine treatment using ^131^I, since the 365 keV gamma rays can lead to a high exposure to other patients, staff and members of the public. In practice this may restrict the more widespread introduction of some therapeutic radiopharmaceuticals based on ^131^I. However, it is important to bear in mind that some gamma emission can be of value for imaging the uptake and biodistribution of the therapeutic agent. Indeed this can be extremely valuable for the assessment of individual patient dosimetry and in planning effective strategies for individual patient treatments. This will allow treatment protocols based on tumour dose prescriptions as performed in external beam radiotherapy [3].

In addition to the physical characteristics of the therapeutic radionuclide, other obvious requirements are that the conjugation of the targeting molecule to the radionuclide should be reliable, practical and affordable. The final radiopharmaceutical conjugate must be suitable for patient administration, stable *in vivo* and effective at targeting the tumour receptor or binding site [4].

## MECHANISMS OF UPTAKE

A number of different mechanisms have been used to achieve selective uptake of radiopharmaceuticals in tissues. These are metabolic processes such as with radioiodine, radiophosphorus and meta-iodobenzylguanidine; the use of specific cell surface receptors for example using radiolabelled hormones, peptides and antibodies and by targeting the extracellular mechanisms using bone seeking agents and radiolabelled cells.

In oncology a feature of tumour growth is the development of a good blood supply to provide the oxygen and nutrients necessary for cellular replication. The new tumour vessels are inherently leaky compared with normal blood vessels. This is due to wide inter-endothelial junctions, large numbers of fenestrae and transendothelial channels formed by vesicles as well as discontinuous or absent basement membranes [5,6]. As a result, capillary permeability of the endothelial barrier in newly vascularised tumours is significantly greater than that of normal tissues [7]. This may lead to increased uptake of some agents since this is a function of both local blood flow and microvascular permeability. The amount of tissue accumulation of a conjugate is proportional to plasma clearance. The enhanced permeability and retention (due to poor lymphatic drainage of the tumour) may lead to prolonged accumulation and retention of macromolecules in tumour interstitium [8].

There have been a number of successes in the field of targeting diagnostic tracers to tumours, since only a relatively small number of sites within a tumour need to take up the tracer for effective imaging. However the targeting of therapeutic radiopharmaceuticals for the treatment of solid tumours is difficult and as such a number of strategies have been employed such as intralesional, intraarterial and intracavitary administration for example as in the treatment of glioma and superficial bladder cancer (see below).

## ESTABLISHED RADIOPHARMACEUTICAL THERAPIES

Three forms of treatment are regarded as the longer running and more established forms of radionuclide therapy. These are ^131^I-sodium iodide for the treatment of thyroid carcinoma and thyrotoxicosis, ^32^P-sodium phosphate for the treatment of proliferative polycythaemia and ^90^Y colloid, ^186^Re-sulphide and ^169^Er-citrate colloid for synovectomy and the treatment of malignant effusions.

### Thyroid

Radioiodine treatment was developed in the 1950s and was one of the very first of the modern nuclear medicine therapies. ^131^I-sodium iodide is available in solution form or as hard gelatin capsule both for oral administration. Once taken, it is absorbed rapidly (90% within the first hour) from the upper gastrointestinal tract. Within the thyroid gland iodine is taken up by differentiated follicular thyroid cells and can be considered unique in nuclear medicine. Radioiodine is used to treat both thyrotoxicosis (toxic diffuse goitre and toxic nodular goitre) and thyroid cancer. Approximately 400 MBq is administered for the treatment of thyrotoxicosis. Thyroid cancer is a relatively common and often curable malignant neoplasm that usually presents with no symptoms other than a lump in the neck, which may be solitary or multinodular. In the United Kingdom, thyroid cancer represents approximately 1% of all malignancy, the annual incidence being reported at 0.9 per 100,000 men and 2.3 per 100,000 women. Thyroid cancer accounts for nearly 2% of all new cancers diagnosed annually in the United States. The treatment options have evolved considerably over recent years however the use of radioiodine remains an important consideration in the practices of both head and neck surgeons and endocrinologists [9]. Surgery (lobectomy or thyroidectomy) is the first choice of treatment followed by ablation with ^131^I. Administered activities of 4 GBq and 8 GBq are used to treat primary and metastatic disease respectively. Extensive use of radioiodine over the past 50 years has proven its efficacy, safety and cost effectiveness. It is remarkable since radioiodine treatment has the benefit of no sickness, no hair loss, no nausea, no diarrhoea and no pain. Radioiodine therapy will remain as one of the main forms of treatment for thyroid disorders for the foreseeable future. Clearly this form of therapy stands as a benchmark against which all new forms of radionuclide therapy can be judged.

### Radionuclide Synovectomy

Radionuclide synovectomy is widely used in many centres throughout the world. Direct injection of radioactivity into the joint spaces is used for the purpose of synovectomy. The radionuclide of choice is dictated by the size of the joint space. Approximately 185 MBq ^90^Y-colloid is used for the treatment of knee joints, where it is estimated that the order of 100 Gy is delivered to the synovium. 74-150 MBq ^186^Re-sulphide is used to treat the hip, shoulder and ankles and small metatarsophalangeal/metacarpophalangeal joints may be treated with 10-40 MBq ^169^Er-citrate colloid. In comparison with surgical synovectomy, radionuclide synovectomy produces comparable results, is less expensive and allows the patient to remain ambulatory. It is often considered the initial procedure of choice for the treatment of patients with hemarthrosis in hemophilia. In addition, local instillation of radiopharmaceuticals can effectively reduce effusions after implantation of prosthesis [10].

### Polycythaemia

Treatment regimens for polycythaemia are usually based on an intravenous injection of 74-111 MBq ^32^P orthophosphate (PO_4_^3-^) per square meter body surface area. ^32^P is incorporated into the nucleic acids of rapidly proliferating cells. The therapeutic aim is to suppress hyperproliferative cells rather than to eradicate them. The clinical use of ^32^P therapy in polycythaemia is variable and the widespread future of this form of treatment is uncertain.

## CURRENT AND EVOLVING RADIOPHARMACEUTICAL THERAPIES

### MIBG

**Figure 1:**
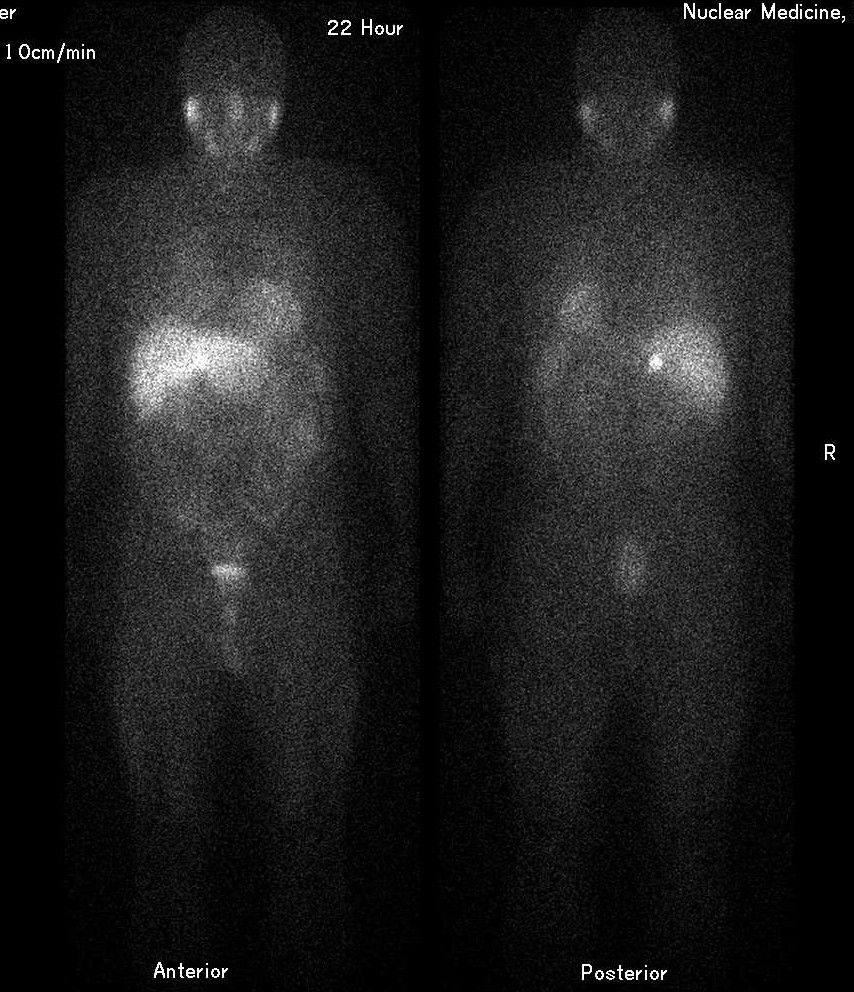
Anterior (left) and posterior (right) whole body scan of a patient with phaeochromocytoma of the right adrenal seen as a small intense focus of uptake. These diagnostic images acquired 22 hours after administration of ^123^I-MIBG can be used to assess the prospects for therapy with ^131^I-MIBG. Uptake of tracer can also be seen in the salivary glands, liver and urinary tract.

Metaiobenzylguanidine (MIBG) is a catecholamine analogue similar to noradrenalin, which accounts for the uptake of this radiopharmaceutical in catecholamine storage vesicles. It is used for diagnostic imaging radiolabelled in the form of ^123^I-MIBG (Figure 1), however when radiolabelled with ^131^I it is used for the treatment of neuroectodermal tumours such as neuroblastoma (which is the second commonest solid tumour in children), phaeochromocytoma and carcinoid. The success of this therapeutic agent has resulted in its use as a first line treatment in metastatic neuroblastoma. Other approaches include the combination of ^131^I-MIBG with chemotherapy and blood stem cell support. The majority of neuroendocrine tumours also possess a high density of somatostatin receptors offering an alternative approach with somatostatin receptor radiopharma-ceuticals [11].

### Radiopeptide conjugates

**Figure 2:**
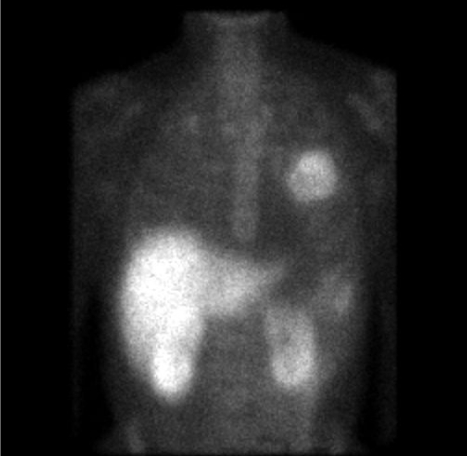
Anterior image of the thorax of a patient demonstrating intense uptake of the 99mTc-labelled somatostatin analog Depreotide (Neospect^TM^) in a solitary large mass in the left lung. High liver and renal uptake can also be seen.

Interest in synthetic peptides has been growing steadily over the past 10 years and a number of radiolabelled agents have a most promising future as agents for targeted radionuclide therapy [12]. Low molecular weight peptides (3,500 Daltons; usually less than 30-40 amino acids in length) have the desirable properties of fast clearance, rapid tissue penetration, and low antigenicity. These molecules can be produced easily and inexpensively and therefore offer an attractive vehicle for clinical use and commercial production. Examples of current radiopharmaceuticals include peptide hormones such as somatostatin, vasoactive intestinal peptide, melanocyte stimulating hormone, oestrogens and progesterone. Of these, somatostatin has proven to be of the greatest interest. Somatostatin is a peptide hormone consisting of 14 amino acids and is naturally present in the hypothalamus, brain stem, gastrointestinal tract and pancreas. The ^99m^Tc-labelled somatostatin analogue, depreotide is a promising tracer for discriminating between malignant and benign lung lesions [13]. An example of the highly positive uptake that can be visualised in patients with small cell cancer of the lung is given in Figure 2. Clearly a tracer having such high uptake would offer potential for targeted therapy, however the high uptake in the liver and kidneys remain limiting factors.

A derivative of somatostatin labelled with ^111^In has been widely used as an imaging agent (Ocreoscan^TM^). Clinical trials have been undertaken with therapeutic amounts of this radiopharmaceutical, however ^111^In is poorly suited for therapy and current studies using ^90^Y are proving to be more promising [14]. In further trials the results obtained with ^90^Y-DOTA(0)-Tyr(3)-octreotide and ^177^Lu-DOTA(0)-Tyr(3)-octreotate are very encouraging in terms of tumour regression [15,16]. Radiolabelled peptides remain as one of the most highly promising vectors for targeted radionuclide therapy.

### Bone seeking agents

The growth of bone metastases involves multiple processes including tumour-cell proliferation, cell-matrix detachment, cell migration, angiogenesis and intravasation. The exquisite uptake of bone seeking agents is evident from the ^99m^Tc-phosphonate bone scan which is one of the most common diagnostic nuclear medicine procedures carried out worldwide. Radionuclide bone scintigraphy with ^99m^Tc is highly sensitive but has variable specificity. The process relies on the detection of an osteoblastic reaction in the presence of bone damage. The sensitivity of ^99m^Tc scintigraphy for detecting bone metastases has been reported to range from 62% to 98%, but false positive rates as high as 40% have been reported [17,18]. Bone scintigraphy is more sensitive and more specific than plain x-ray films and computed tomography (CT), while magnetic resonance imaging (MRI) is considered by some to be superior to bone scan in evaluating vertebral metastases.

The high uptake of bone seeking agents has led to the widespread use of beta emitting agents for the palliation of painful bone metastases from carcinoma of the prostate and breast [19,20]. The main radiopharmaceuticals available include ^153^Sm-EDTMP, ^89^Sr-chloride, ^153^Sm-EDTMP, ^186^Re-HEDP and ^117m^Sn(IV)-DTPA. ^89^Sr and ^153^Sm radiopharmaceuticals are the most widely approved radionuclides for the palliation of pain from metastatic bone cancer throughout U.S., Europe and Asia. Radiopharmaceutical treatments generally provide effective pain relief with response rates of between 40% and 95%. Pain relief starts at between one to four weeks after the initiation of treatment and continues for up to 18 months and reduces analgesic use in many patients. Mild and reversible thrombocytopenia and neutropenia are the most common toxic effects. Continued pain relief may be achieved in many patients by repeat administration. The effectiveness of radiopharmaceutical therapy can be increased when combined with chemotherapeutic agents.

A particularly promising conjugate for the future is based    on the ^188^Re produced from the tungsten generator system. ^188^Re is in the same periodic group as ^99m^Tc and the tungsten generator has many similarities to the molybdenum generator, with the potential of becoming its therapeutic equivalent for the production of a wide range of therapeutic radiopharmaceutical conjugates. Liepe *et al.* [21] have demonstrated ^188^Re-HEDP to be an effective radiopharmaceutical used in the palliative treatment of metastatic bone pain in prostate cancer with minimal bone marrow toxicity. In this study 76% of the patients treated described relief of bone pain without increase of analgesic intake.

In the future radiopharmaceutical treatments may become of important clinical value for the therapy of bone metastases rather than palliation. Some studies with ^89^Sr and ^153^Sm have indicated a reduction of hot spots on bone scans in up to 70% of patients suggesting a possible tumouricidal action. Further clinical trials are needed to determine the optimum combination of radionuclide conjugate and dose schedule for effective therapy.

### Radioimmunotherapy

The concept of using antibodies to deliver substances to tumour cells was first suggested by Paul Ehrlich in 1896 when he coined the term “magic bullets”. A ‘vehicle’ with specificity for a receptor expressed solely on malignant cells serves as a carrier molecule for a cytotoxic agent. Delivery of the radionuclide to tumour cells causes specific cell killing while sparing normal cells the cytotoxic effects. The specificity of the antibody-antigen interaction is indisputable therefore antibodies are powerful targeting molecules. The development of monoclonal antibody technology in 1975 by the two research scientists Kohler and Milstein in England has led to the production of large amounts of antibody of reproducible and precise specificity. This resulted in a dramatic increase in interest in the field of antibody-targeted therapeutics. In the early 1980s, there was a general belief that Ehrlich's theory was close to being realised with early publications showing excellent sensitivity and specificity for the *in vivo* detection (immunoscintigraphy) of colon and ovarian cancer and melanoma [22-25]. This raised the possibility that antibodies could be used, not only to detect cancer but also to treat it. Over the past 20 years there has been a great deal of work on the construction of antibody conjugates of different molecular designs [26] and incorporating different radiolabels [4]. Much progress has been made and licensed therapeutic products are now emerging for clinical use.

**Figure 3:**
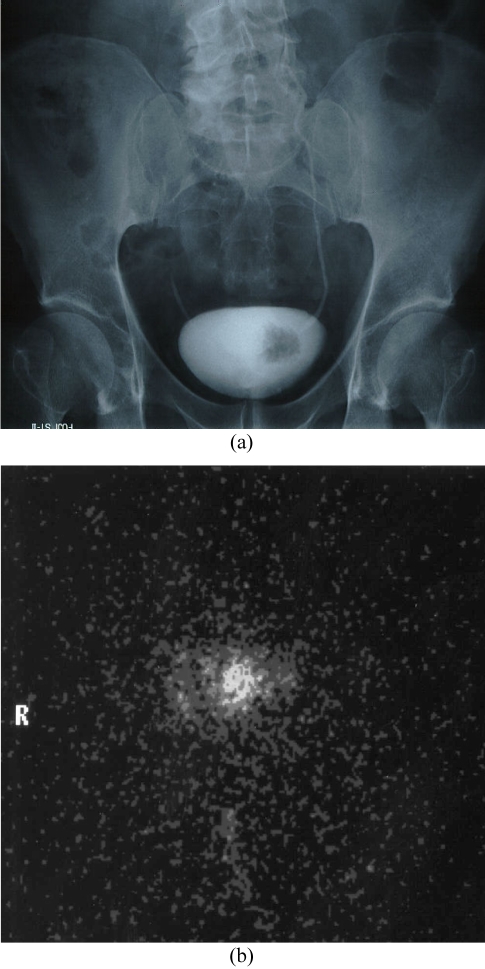
(a) Intravenous urogram showing large superficial transitional cell tumour in the left side of the bladder; (b) anterior gamma camera image showing uptake of ^188^Re-C595 anti-mucin antibody, following intravesical administration for 1 hour.

In order for radioimmunotherapy to be successful the tumour must be accessible to the immunoconjugate through the chosen route of administration. Intravenous injection is the most common form of administration. However systemic administration of an immuno-conjugate can result in sensitisation of the host immune system, particularly if the antibody is of murine origin. This can result in the formation of human anti mouse antibody (HAMA) that can cause increased clearance of subsequent treatments and may even cause serious anaphylactic reactions in the patient. Another problem with systemic administration is the potential toxicity to organs such as the kidneys and liver that would normally clear such macromolecules from the circulation. One approach that limits the problems posed by HAMA response and hepatic and renal toxicity is to administer the immunoconjugate locally to the site of the tumour. This can be achieved in some cases by injection directly in to the tumour mass. This approach has been taken for the treatment of some brain tumours such as glioma [27]. In addition, some tumours such as those in the bladder, grow in body cavities that shield them from the host immune system. Intravesical antibody targeted therapy provides advantages over systemic administration in that no HAMA response is generated and higher doses of the therapeutic agent can be given without causing problems of non-target organ toxicity. Phase I studies have been undertaken with ^67^Cu-labelled anti mucin antibody for the treatment of superficial bladder tumours [28]. However ^67^Cu is not widely available and one interesting possibility is the use of ^188^Re-labelled antibody for this application [29]. An example of the intravesical targeting of antibody to bladder tumour from the author's work is shown in Figure 3. It is obvious however that only a limited number of tumour types fulfil the criterion for intravesical therapy and this route of administration will not allow efficient targeting of distant micrometastases.

The first therapeutic antibody products to attract commercial interest were for the treatment of diffuse haematological malignancies [30]. In 1997 the first antibody conjugate to be approved by the FDA for targeted cancer therapy, was an anti-CD20 antibody (rituximab) for the treatment of Non-Hodgkin's Lymphoma (NHL). NHL is inherently sensitive to radiation and is one of a number of haematological malignancies that can benefit from radioimmunotherapy [31]. Radiotherapy can be curative in early stage NHL but is less easily applied to advanced stage disease. One attractive feature of radiopharmaceutical therapy is that there appears to be a synergistic activity between naked antibody and the radionuclide. Prospective trials comparing ^90^Y-ibritumomab tiuxetan with single-agent rituximab are showing an overall response rate (ORR) to ^90^Y-ibritumomab tiuxetan of 80% with 34% complete response. Similar results have been reported with ^90^Y-ibritumomab tiuxetan in patients with relapsed or refractory low-grade NHL with mild thrombocytopenia. Safety data from patients entered into studies have demonstrated side effects that are mainly hematological and transient [32,33]. Initially two licensed products were commercially available, Zevalin^TM^ (ibritumomab tiuxetan) and Bexxar^TM^. Both antibodies react with the CD20 antigen, which is important for cell cycle initiation expressed only on B-lineage cells and is considered to trigger apoptosis. Bexxar^TM^ is radiolabelled with ^131^I whereas Zevalin^TM^ has ^90^Y as the therapeutic radionuclide. Clearly, this will affect the clinical utility of the product since Bexxar^TM^ may be used for imaging to assess treatment planning, uptake and dosimetry, wheareas Zevalin^TM^ is suitable for outpatient administration. It will be interesting to see how widespread these therapeutic radiopharmaceuticals are used in future.

## FUTURE PROSPECTS

The fundamental concept of external beam radiotherapy is the delivery of a cancerocidal dose of 60-70 Gy to tumour tissue without critical damage to healthy organs. In practice the treatment of disseminated cancers is limited to 30-40 Gy in order to spare normal tissue damage. It is with this promise of minimising irradiation of normal tissues that radionuclide therapy stands to gain future prominence. Recent studies using functional metabolic tracers such as FDG are beginning to show fundamental errors in the definition of tumour volume based on purely anatomical imaging such as x-ray CT [34]. In the same way that FDG can demonstrate functioning tumour tissue, the targeting of a more potent radionuclide to molecular functioning tumour tissue should offer an advantage in the treatment of a wide spectrum of pathologies. Whereas therapeutic radiopharmaceuticals based on ^131^I-sodium iodide for the treatment of thyroid disease and ^131^I-MIBG for neural crest tumours are well established with an assured role in the foreseeable future, the use of radionuclides for polycythaemia and synovectomy are less certain.

Radiopharmaceutical treatments of the future will certainly employ antibodies, peptides and labelled drug molecules, however there are still some new technologies to be developed for use in nuclear oncology. Dramatic growth in the biotechnology sector has led to new techniques for the design, selection and production of ligands capable of specific molecular recognition. These alternative molecular entities can be generated rapidly to recognise molecular targets such as tumour-associated antigens. One promising approach is the production of receptor binding molecules based on specific nucleic acid sequences that are capable of recognising a wide array of target molecules. These oligonuclide ligands are known as aptamers [35,36]. The term aptamer is derived form the Latin word “aptus” meaning “to fit”. The technology that allows production of aptamer molecules is known as systematic evolution of ligands by exponential enrichment (SELEX). These molecules offer the prospect of good tumour penetration, rapid uptake and clearance, thus providing effective vehicles for cytotoxic agents. Manipulation of the molecular weight of these constructs utilising methodologies to produce polymeric aptamer complexes should achieve an optimum balance between rapid renal clearance that leads to premature elimination of the complex from the system and adequate tumour uptake for diagnostic imaging and targeted therapy.

The choice of the optimum radionuclide is still to be determined although ^90^Y appears to be emerging as an important therapeutic label. In future, it is highly likely that ^188^Re from the tungsten/rhenium generator will play an important role, particularly as this provides a reliable source of a therapeutic radionuclide over a sustained period of up to 6 months from one generator, this being of particular value to remote and developing countries wishing to benefit from these procedures.

Another exciting area of interest is the use of conjugates based on alpha emitters such as ^211^At, ^212^Bi and ^212^Tb [37]. These radionuclides have energies of an order of magnitude greater than beta emitters having linear energy transfer of about 100 times greater than beta particles giving them a range of 50-90 mm in tissue. Typically absorption of alpha particles can result in 0.25 Gy in 10 mm cell diameter. *In vitro* studies show that alpha therapy is more cytotoxic by one to two orders of magnitude to targeted cells than non-specific alpha conjugates, specific beta emitting conjugates or free radionuclides [38]. Currently these forms of therapy are entering Phase I and II trials for treatment of melanoma, leukaemia and tumours of breast, pancreas, and prostate. Initial results from clinical trials of ^223^Ra given as radium chloride at doses of up to 350 kBq/kg for palliation of metastatic bone pain from prostate carcinoma are also showing potential value [39].

In conclusion it is evident that the future of molecular targeted radionuclide therapy is assured and this modality will prove to be a valuable therapeutic tool of particular benefit in clinical oncology. Although at present largely based on ^131^I and ^90^Y, the choice of therapeutic radionuclide is widening to include generator products and possibly alpha emitters. Treatment regimes will be developed to employ dose fractionation to allow outpatient administration. However, the routine application of therapeutic radiopharmaceuticals is likely to be limited to specialised centres with the expertise, facilities and authorisation for handling large amounts of radioactivity.
